# Use of Alcohol and Unprescribed Drugs after Suicide Bereavement: Qualitative Study

**DOI:** 10.3390/ijerph16214093

**Published:** 2019-10-24

**Authors:** Jessica Eng, Lauren Drabwell, Fiona Stevenson, Michael King, David Osborn, Alexandra Pitman

**Affiliations:** 1 UCL Division of Psychology and Language Sciences, 26 Bedford Way, London WC1H 0AP, UK; j.eng.17@alumni.ucl.ac.uk; 2 UCL Centre for Behaviour Change, Research Department of Clinical, Educational, and Health Psychology, University College London, 1-19 Torrington Place, London WC1E 7HB, UK; lauren.drabwell.17@ucl.ac.uk; 3 UCL Research Department of Primary Care & Population Health, Rowland Hill St, London NW3 2PF, UK; f.stevenson@ucl.ac.uk; 4 UCL Division of Psychiatry, Maple House, 149 Tottenham Court Road, London W1T 7NF, UK; michael.king@ucl.ac.uk (M.K.); d.osborn@ucl.ac.uk (D.O.); 5 Camden and Islington NHS Foundation Trust, St Pancras Hospital, London NW1 0PE, UK

**Keywords:** suicide, bereavement, grief, alcohol, drug, qualitative research, thematic analysis

## Abstract

Studies describing the impact of suicide bereavement report an excess risk of suicide, suicide attempt, psychiatric illness, and drug and alcohol use disorders compared with the general population. However, the nature of patterns of drug and alcohol use after suicide bereavement is unclear. We used an online survey to collect qualitative data to understand whether and how drug and alcohol use changes after suicide bereavement. We conducted thematic analysis of free-text responses to a question capturing their use of alcohol and drugs after the suicide of a family member or a close friend. Analysing data from 346 adults in Britain aged 18–40, we identified three main themes describing the relationship of suicide bereavement to alcohol or drug use: (1) control over drug or alcohol use, (2) the perceived purpose of using drugs or alcohol, and (3) the attribution of drug or alcohol misuse to external factors. Overlying these themes were dimensions of control and of awareness of potential harms. This study highlights that increased use of drugs and alcohol after suicide bereavement may form part of a bereaved person’s coping strategies, and that sensitive approaches are needed when judging whether and when to intervene.

## 1. Introduction 

Approximately 800,000 people die by suicide annually worldwide [1], and suicide is the second leading cause of death for young men internationally [2]. Empirical estimates suggest that each suicide has emotional effects on between 60 [3] and 135 [4] friends and family members. Suicide bereavement describes the adjustment, grief and mourning period after a suicide [5] and has a number of adverse health and social outcomes. Studies describing the impact of suicide bereavement report an excess risk of suicide, psychiatric illness, and drug and alcohol use disorders compared with the general population [6]. Parental functioning, communication, and familial cohesion can be impaired within families who experience suicide [7,8]. Children who experience suicide bereavement are more likely to struggle at school and to become diagnosed with mental health disorders such as depression and anxiety [8]. For these reasons, it is important that people bereaved by suicide receive the right support to mitigate these potential adverse effects. However, survey research suggests that people bereaved by suicide are less likely to receive timely informal support than those bereaved by other causes of sudden death, perhaps due to the stigma of suicide [9]. It is also important to understand why some people who experience suicide bereavement struggle with mental illness, social disruption and suicidal thoughts, so that we can develop effective interventions by which to support them.

It is possible that misuse of alcohol and drugs might contribute to mental health and social difficulties. The links between alcohol and drug use and mental health problems are well established [10], but not well understood in relation to bereavement. Studies of people bereaved by any cause suggest that alcohol consumption and drug use may increase after bereavement [11], perhaps as a way of coping with loss and loneliness. Comparing suicide bereavement with other losses, the evidence suggests that substance misuse may be more characteristic of suicide bereavement in children but not in adults. A Danish registry-based study found an excess risk of being diagnosed with an alcohol or drug use disorder in spouses bereaved by suicide compared with non-bereaved controls, but not when compared to spouses bereaved by other causes [6]. A Canadian registry-based study found higher rates of alcohol and drug use disorders in suicide-bereaved parents compared with non-bereaved parents, but no differences when compared with parents bereaved by a child’s road traffic death [12]. The same study also found no increase in alcohol or drug misuse disorders in suicide-bereaved parents when comparing the periods before and after the loss, but this was probably explained by their higher risk of alcohol and drug use disorders even before their child’s suicide [12]. A Swedish registry-based study found that young people bereaved by parental suicide were at no greater risk of admission for alcohol or drug use disorders than young people bereaved by other causes of parental death [13]. However, this study also found that suicide bereavement in childhood was associated with a higher risk of hospitalization for drug disorders compared with those who lost a parent to suicide later in development [13]. A longitudinal study in the USA comparing bereaved young people with their non-bereaved peers found that the former were at greater risk of alcohol and drug misuse or dependence, especially adolescent boys with disruptive behavior disorders [14]. However, these risks were similar whether the bereavement was due to suicide or other sudden causes [14]. Other longitudinal research in the USA has found that suicide-bereaved youth had a higher risk of developing an alcohol or drug misuse disorder compared to youth bereaved by other mortality causes [15].

The above studies rely on a recorded clinical diagnosis of alcohol or drug misuse, which in turn relies on the problem being identified by health services. Given the spectrum of alcohol and drug use patterns that lie beyond a formal diagnosis, a more fine-grained approach is required to understand how traumatic bereavement might affect drug or alcohol use. Cross-sectional survey research in the USA has found that adolescents bereaved by a relative’s suicide were more likely to self-report marijuana use and binge drinking compared to unexposed adolescents [16], whilst those exposed to a friend’s suicide were more likely to self-report smoking, marijuana use, and binge drinking than unexposed peers [8]. More studies are needed using self-report measures and qualitative approaches in order to understand in greater detail how drug or alcohol use might change after traumatic bereavement. Our aim was to use qualitative approaches in analysing data collected from an anonymous online survey to gain a more nuanced understanding of any changes in use of alcohol or drugs after a suicide bereavement, and the motivations behind any changes.

## 2. Materials and Methods

### 2.1. Methodological Approach

Our research questions were whether suicide bereavement influences use of alcohol or illicit drugs, and what the nature of any such changes are. To answer these we conducted an inductive analysis of responses to a specific question within a wider survey, acknowledging an awareness of the potential perceived harms and benefits of alcohol use.

### 2.2. Participants and Design

We conducted a national cross-sectional closed online survey to collect qualitative responses to a question on use of alcohol and drugs from a nested sample of people bereaved by suicide, drawn from a wider sample of people bereaved by sudden death. The sampling methods for this study—the University College London (UCL) Bereavement Study—have been described previously [5,17,18,19]. In summary, we invited all 164 higher education institutions (HEI) in the UK in 2010 to participate in our online survey to investigate the impact of sudden bereavement. A total of 37/164 (23%) of HEIs agreed to participate by sending all their students and staff individual email invitations with an embedded survey link. Due to the sensitivities of the topic, 10 HEIs chose to modify this by advertising the study on their weekly email digests, advertising the survey on both the student and staff intranet, or sending the survey solely to students. HEIs represented a range of agricultural, arts, and academic institutions, providing broad geographic and socioeconomic representation.

Our inclusion criteria were respondents aged between 18 and 40 who had experienced the sudden bereavement of a close friend or relative since the age of ten. Participants self-reported their exposure to suicide, sudden natural causes (e.g., coronary death) and sudden un-natural causes (e.g., accidental death). Our exclusion criteria were exposure to bereavement before the age of ten, in order to reduce recall bias.

The questionnaire consisted of 119 closed questions on socio-demographic and clinical characteristics of participants and 20 open questions addressing specific aspects of bereavement such as impact on occupational functioning, relationships, and religiosity (supplementary 1). The questions aimed to be neutral and non-leading to avoid the assumption of solely negative outcomes after bereavement. There was no word limit on free text answers and participants were requested to provide as much or as little information as they wanted, or to skip the question if it did not apply. Once they finished the survey, all participants were directed to a list of support services.

The current analysis focused only on people exposed to suicide bereavement, and their responses to the question: “In what way, if any, has the bereavement affected your drinking habits or your use of unprescribed drugs? Unprescribed drugs include illicit drugs as well as medications used above their prescribed limits”. In wording this question we chose to use the term unprescribed drugs to capture illegal drugs and the abuse of prescription and over-the-counter medications. This was also the wording suggested by our consultation group of young bereaved adults and bereavement counsellors, and that indicated when piloting the questionnaire nationally [17].

### 2.3. Ethics

The UCL Research Ethics Committee approved this study in 2010 (ref: 1975/002), and all participants provided their consent online. They were additionally informed that the data would be handled in accordance with the Data Protection Act (2010).

### 2.4. Analytic Approach

We used thematic analysis of the free text responses arising from this open question [20]. We imported data into Microsoft Excel, which allowed us to organize, review, and code large volumes of relatively short free text responses, by adding columns for successive rounds of coding and categorizing. We took a collaborative approach as a group of researchers (Jessica Eng, Lauren Drabwell, and Alexandra Pitman), reflecting a range of perspectives (clinical psychology, health psychology, and psychiatry respectively).

First, we identified those who provided uninterpretable responses, and excluded these to confine our analysis to responses for which meaning could be interpreted. Having reviewed all responses to facilitate immersion in the data we then conducted in-depth content-based coding, developing codes and themes to capture aspects of respondents’ experiences. In the first stage of the analysis, two researchers (Jessica Eng and Lauren Drabwell) coded a sample of data from 100 participants independently, in order to develop independent data-driven coding frameworks. As a team we then compared these codes to agree on the initial coding framework and resolve any discrepancies. Data analysis progressed through further cycles of coding and reflective team discussions, revisiting the data repeatedly to check the consistency and face validity of codes. During this process we continually revised our coding framework by collapsing codes and developing new themes, sub-themes, and higher-order themes. We used regular meetings to explore any differences in interpretation, challenging each other over any apparent preconceptions derived from team members’ differing perspectives. This reflexivity enhanced the validity of our findings, as did our final review of the original data against the final coding framework.

We followed COREQ (Consolidated criteria for Reporting Qualitative research) guidelines to structure our findings [21]. Typographic errors were corrected where mistakes was obvious e.g., where letters were omitted or inverted.

## 3. Results

### 3.1. Response

Overall, 652 of the 5087 respondents to the survey invitation indicated a past history of suicide bereavement. Of these, 347 (53%) responded to the question on substance use. We excluded 1 participant who responded ambiguously as “*anti-anxiety medication*”. Our final analytic sample consisted of 346 participants who provided free text responses describing the impact of suicide bereavement on use of drugs or alcohol. Of these, 137 indicated briefly (“*not applicable*”; “*no change*”) that their use of alcohol or drugs had not changed since the bereavement, but without expanding further.

### 3.2. Participant Characteristics

Of our sample of 346 participants, mean age of participants was 25.6 (standard deviation = 5.99). The majority of the sample were white, young, educated females (Table 1). Their kinship to the deceased was as follows, in order of frequency: close friend (107; 31%); father (54; 16%); brother (34; 10%); cousin (27; 8%), uncle or aunt (26; 8%), partner/spouse (16; 5%), ex-partner (14; 4%), other (14; 4%); sister (10; 3%); grandparent (7; 2%), colleague/client (4; 1%); missing (5; 2%), niece/nephew (3; 1%), and brother/sister/mother/father-in-law (2; 1%).

### 3.3. Thematic Analysis

In their free text responses participants indicated a range of patterns of use. These included sustained increased use and/or decreased use, transient increase in use immediately after the bereavement followed by a decrease in use, and no change in use but a shift in attitudes towards drugs or alcohol. In our analysis of any changes in use of alcohol or drugs after a suicide bereavement, and the motivations behind any changes, we identified three overarching themes. These were: (1) control over drug or alcohol use, in which participants described the difficulties they had, or efforts they went to in controlling their use; (2) the perceived purpose of using drugs or alcohol, in which participants described harnessing specific functions from their use of either; and (3) the attribution of drug or alcohol misuse to external factors, in which participants ascribed an increase in use to life transitions or peer influences (Table 2).

#### 3.3.1. Theme 1: Control over Drug or Alcohol Use

Many participants described how they felt they had lost control over drug or alcohol use after the suicide, or that they had had to make deliberate efforts to control their use. For those who felt they were using drugs or alcohol above recommended limits it was clear that they saw this as a problem, with potentially harmful effects. For those who limited their use, this was out of a fear of the effects of drugs or alcohol on their emotions or their health, and in some cases a fear of this leading to their own suicide.

(1) Loss of control over drug or alcohol use. Many participants were aware of having lost control of their substance use since the suicide, for varying periods of time. The terms they used (“*stupid amounts*”; “*binge drinker*”; “*out of hand*”; “*I had a problem*”) indicated an awareness of the harmful effects of the behaviour.

“I cannot go out anymore for a couple of drinks instead I drink stupid amounts” (30 years old female, 2 years since close friend’s suicide)

“Before the death I would hardly ever drink. In the 2 months after the death I drank every day and began smoking-again something I never did and hated. I have since tried drugs and was entirely against the use of them before the bereavement.” (20 years old female, 5 months since close friend’s suicide)

A number of participants looked back on this as a distinct period after the loss, following which they had either regained control or no longer needed this intoxication. The implication was that daily or binge drinking or drug misuse had served a purpose over that period, but then the benefits had ceased to outweigh perceived harms.

“I was pretty much drunk for 3 or 4 months last year, wound up in AA. Since then though I found my mood switched again and I’ve barely drunk alone or in large amounts since.” (20 years old female, 6 years since father’s suicide)

“I went through a stage of drinking almost every evening. I have since dealt with this but the worst thing about it was I would drink at home as opposed to going out with friends and having ‘a good time’” (23 years old female, 12 years since mother’s suicide)

(2) Restraint over use of drugs or alcohol. In contrast to those who perceived a loss of control, a separate group of participants described how they controlled their use of substances by consciously limiting it. Many reported that the bereavement had changed their attitudes towards drugs and alcohol. They were either more conscious of not allowing themselves to become intoxicated or had set specific restrictions on their substance use. Such restraint was often described as having arisen from an awareness of having lost control over drug or alcohol use in the immediate aftermath of the death. This sub-theme therefore overlapped with the previous sub-theme describing transient excessive use.

“For a year after I didn’t drink because I didn’t want to use alcohol to solve a problem since I knew this would only make it worse. My brother who was 16 then, however, did increase his drinking.” (21 years old female, 2 years since father’s suicide)

“stopped taking drugs altogether. Do not drink as heavily as feel too overwhelmed now when I do, (I) get anxiety and an urge to want to keep drinking so I now avoid drinking heavily as much possible. (30 years old female, 1 year since close friend’s suicide)

Sometimes the reported motivation for reining in substance use was a fear of losing control over emotions or over continued use. Some described an awareness of being able to feel their emotions more when they were drunk or high, but finding this overwhelming. The fear of becoming engulfed by emotions, or of disclosing hidden feelings to peers, lay behind efforts to restrict use. This lay in contrast to theme 2 below, in which other participants used drugs or alcohol to numb their emotions or escape from them.

“For 2 years after the suicide I stopped drinking alcohol as it made it harder for me to control my emotions.” (27 years old female, 3 years since ex-partner’s suicide)

“I have got extremely drunk accidently since the death and ended up crying and running away from people I am with to be on my own, although I only vaguely remember these nights. I sometimes feel like drinking more than normal, but sometimes avoid it for fear that hidden feelings may come out while drunk.” (21 years old female, 6 months since grandfather’s suicide)

For some participants their restraint over use of alcohol or drugs related to a strong aversion to its use, seeing this as associated with the suicide. In some cases this involved avoidance of the substance used in overdose. For others, notably those bereaved due to parental suicide, it was more indirect and related to their experiences of growing up with an alcoholic parent. Although it was not always clear whether alcohol was implicated in their parent’s suicides, the avoidance of alcohol seemed to relate to an aspiration that their own life would not “*turn out like that*”.

“My cousin was found with weed in his system, so actually I think I have developed a fear of drugs and avoid them more than I perhaps would have.” (22 years old female, 10 years since cousin’s suicide)

Active avoidance was not true for all respondents for whom alcohol misuse had particular resonance. One 22 years old woman noted that her alcohol intake was unchanged since her father’s suicide four years previously, and that this was “*quite ironic considering my father died from alcohol poisoning*.”

#### 3.3.2. Theme 2: Perceived Purpose of Using Drugs or Alcohol

Many participants were clear that they used drugs or alcohol for a specific purpose, and therefore as a coping mechanism. This theme was broadly divided into those who had increased their use of drugs or alcohol to block out emotions, to relax or to sleep, and those whose intake had not changed but for whom drinking took on a special significance in relation to the deceased.

(1) Coping with overwhelming thoughts and emotions. Many participants described using alcohol or drugs to cope with the emotions they were experiencing, either to reduce their impact by blocking them out, or to escape from them. This contrasted with those who used drugs or alcohol to slow thoughts down to a manageable pace, as described below. Participants who did not want to feel anything after the bereavement used substances as a conscious means of blocking emotions, to avoid feeling overwhelmed.

“I used to drink every night when I got old enough to buy alcohol. I couldn’t bear to feel like I did, so alcohol took those bad feelings away. Drugs also helped to numb things” (36 years old female, 25 years since grandfather’s suicide)

“I drank a lot of alcohol for a long time after the death-it was easier not to feel anything.” (36 years old female, 15 years since partner’s suicide)

*“I probably drink far too much, and I drink for the wrong reasons* i.e., *to block things out and to get out of my head-probably not healthy.” (21 years old female, 10 years since mother’s suicide)*

Whilst many reported using alcohol and drugs to cope with their emotions, a small minority of participants described using substances to relax and specifically to help them sleep. This was more apparent in relation to drug use. Achieving relaxation either meant feeling a release or slowing down one’s thoughts. Those who suffered from insomnia used drugs to slow down their thoughts and relax in order to help them sleep. The accounts of some suggested that in the longer-term their drug use had led to apparent dependence.

“started smoking as a definite result of my feelings as it relaxed me, which was hugely useful at the time although I haven’t stopped since.” (21 years old male, 3 years since close friend’s suicide)

“Following the bereavement, I used cannabis frequently (avg x5 weekly). This helped my thoughts to slow down and enabled me to sleep (finally). I was prescribed zopiclone, but this only worked when taken with alcohol.” (36 years old female, 10yrs since partner’s suicide)

(2) Honouring the memory of the deceased. A few participants reported no change in their use of alcohol and drugs after the bereavement but described how they regarded alcohol or drug use as a way to emulate or honour the deceased. This was more apparent in relation to alcohol use. By engaging in behaviour characteristic of the deceased, such as drinking their favourite drink or going out to party, they felt they were able to respect their memory.

“Hasn’t affected this. We tend to drink a wine [the deceased] would have liked when we meet up but not altered the volume at all.” (35 years old female, 11 months since close friend’s suicide)

“I drink as much as before, I’ve never drunk in stupid amounts. He was a big partyer so I go out in memory of him.” (20 years old female, 8 months since close friend’s suicide)

#### 3.3.3. Theme 3: Attribution of Drug or Alcohol Misuse to External Factors

Although most people acknowledged some agency over their use of drugs or alcohol, a minority of participants attributed their increased use to external influences, particularly the prevailing peer culture. The social acceptability of heavy drug or alcohol use in local peer groups, particularly when transitioning to new social groups at university or at certain times of the year (Christmas, exams), was viewed as highly influential on substance use.

“I did binge drink a lot around the time of my friend’s death and continued to do so for the next few years but that period also coincided with going to university so may have not been connected to the bereavement” (38 years old female, 21 years since close friend’s suicide)

“It might have marginally increased my use of weed at the time, and as I had finals that year this might not have helped.” (30 years old male, 8 years since close friend’s suicide)

It seemed it was hard for respondents to judge the extent to which the bereavement had affected their drug or alcohol use in the context of these social influences and life transitions. It was also hard to interpret the extent to which respondents were in denial about their own agency over substance use, suggesting a tendency to an external locus of control.

## 4. Discussion

### 4.1. Main Findings

Our analysis of qualitative data describing the impact of suicide bereavement on use of drugs and alcohol identified a variety of patterns in substance use following the loss. The majority of participants described a change in their use; whether increased or decreased, transient or long-standing. We also identified a broad range of rationales for such changes in use, as captured in our three main themes. Overlying these themes was a tension between two dimensions: degree of control over use, and awareness of the potential for harm. The latter was apparent in participants expressing concerns about the degree to which they were relying on substances, an awareness of the health consequences, and of the effects on their emotions. The dimension of control was manifested in our first theme through descriptions of difficulties in limiting use, and also deliberate restraint, and in the second theme through a wish to avoid the harms that may have contributed to their loved one’s suicide. Restraint was often due to a fear of perceived harms to health or dignity. Some felt in control of their alcohol or drug use in serving as relaxants, hypnotics, or as a way to escape from their emotions, or remember the deceased. The perceived value of drugs or alcohol in serving these roles was balanced against an awareness of potential harms. However, without specifying amounts consumed it was impossible to assess how many were using alcohol or drugs above recommended limits yet not perceiving this as a problem. Those who blamed peer influences or key life transitions for increases in drug or alcohol use placed the problem elsewhere, but the co-occurrence of bereavement and these social transitions made it harder to discern exactly which of them the substance use was attributable to. Across our dataset the tension between efforts to control substance use and an awareness of potential harms is likely to account for the descriptions of the transient nature of excessive drug or alcohol use for some, as reported across all themes.

We observed some variation in themes by age and pregnancy status. Some of those who had been below 18 at the time of the loss had used alcohol or drugs despite being under the legal drinking age, whilst others explained that being underage had restricted their use of alcohol as a coping mechanism. Those who had been pregnant at the time of the bereavement described this as having restricted use of alcohol and drugs and were able to control their use. We did not observe any variation in themes by gender, but the low proportion of men in the sample (19%) may have hampered detection of gender differences.

### 4.2. Results in the Context of Other Studies

Little qualitative research has been conducted exploring alcohol or drug use after suicide bereavement. One Australian interview study with parents bereaved by a child’s suicide identified excessive alcohol as a coping strategy used by some to avoid the pain of thinking about the loss, or as a tool used to help them sleep [22]. These findings parallel those in our British study. Other British and Australian qualitative interview studies exploring the changes suicide-bereaved individuals perceived after their loss did not identify drugs or alcohol as key issues [23,24]. An Australian interview study with suicide-bereaved adolescents quoted one participant who felt that young people lacking informal support or other coping mechanisms might turn to illicit drugs, but this was not a prominent theme [25].

### 4.3. Strengths and Limitations

To our knowledge this is the only qualitative study to use a direct question to all participants probing their use of drugs and alcohol after sudden loss. Using online data collection, we were also able to sample a large and representative sample of young people studying or working in British higher educational settings, whilst avoiding using a solely help-seeking sample. The anonymity of our online data collection favoured disclosure of sensitive material regarding behaviour that might be regarded as undesirable, shameful and/or illegal. The survey question we used amalgamated use of alcohol and drugs, but as motivations behind use of each might differ, we lacked an understanding of the comparative attitudes towards each in terms of balancing utilities and harms. The limited details presented on precise substances used made it hard to distinguish between attitudes to use of alcohol, illegal drugs, over-the-counter medications, and prescribed medications.

We acknowledge that our online sampling methods may have given rise to biases [26]. Firstly, only 20% of our sample was male, representing male non-response bias. By sampling from HEIs we introduced selection bias by favouring those in higher socioeconomic groups. Our inclusion criteria also restricted sampling to those aged 18 to 40. Our findings are therefore more representative of white, highly educated, young women. This is important because of the strong cultural influences on drug and alcohol use. Our collaborative team approach kept to the fore our awareness of researcher influences on coding, and in considering reflexivity we enhanced the validity and consistency of our coding framework. Some responses were ambiguous, and without interviewer presence it was impossible to probe meaning, clarify quantities or frequencies of use, or indeed to respond to distress if this arose. We also lacked objective measures of current and past use in which to contextualise the accounts of participants. We did not compare the accounts of those bereaved by suicide with those of people bereaved by other causes of sudden death, so were unable to ascertain whether our themes were specific to this group. However, we are currently analysing responses to the same question on substance use as posed to people bereaved by other sudden causes, and those findings will serve as an important comparator.

### 4.4. Clinical, Policy and Research Implications

Our key clinical finding was that it was common for people to increase their use of alcohol and drugs after a suicide loss, even if transiently, and that many showed an awareness that substance misuse was not a helpful way to cope with bereavement. Some struggled greatly to control their use, and perhaps would have benefited from specific support at this time, whether to find more adaptive coping mechanisms or to access help with substance misuse. Others viewed drugs and alcohol as fulfilling an important role, and might have regarded intervention as inappropriate, despite having an awareness of the harmful effects. This study gives clinicians an insight into the difficulties that bereaved people cope with, the extent of substance use as self-medication for these problems, and the degree to which intervening is likely to be acceptable. A clinically important sub-group of participants used drugs or alcohol to help them sleep. Many people are prescribed sedatives or antidepressants by doctors to help them cope with severe bereavement, despite evidence that talking interventions are more effective in reducing depression and self-harm [27]. Unfortunately, the benefit to harm ratio is low for all such substances and the dangers not always apparent to the bereaved. For general practitioners (GPs), inviting a bereaved person in for regular reviews, yet holding off on offering interventions to reduce substance use, may be the best way to allow a bereaved person to process the loss. Knowing when to intervene before coping behaviour becomes hazardous is a key clinical skill and relies on a good rapport. At this point, there may be a role for brief interventions delivered in primary care to reduce heavy drinking, as supported by a Cochrane review [28].

The policy implications of these findings are that future commissioning of suicide bereavement support services may need to include screening for harmful use of alcohol or drugs, and address this in a holistic manner. It is particularly important that bereaved individuals are offered help when they need it, given the elevated risk of suicide in this group [6], and the disinhibitory effect of drugs and alcohol. Bereavement counsellors, and others offering support to the suicide-bereaved, may need to address this by exploring other coping mechanisms, as well as in signposting to GP consultation or to substance misuse services. Addressing transient binge drinking or drug use within the suicide bereavement support setting may be more acceptable to the suicide-bereaved, avoiding further stigmatisation and retaining a therapeutic relationship with a professional who understands the context of suicide loss. Support guides developed for people bereaved by suicide [29] should also explain that use of drugs or alcohol as a coping mechanism can sometimes become problematic, explaining where to seek help. They may also describe other coping mechanisms found to be helpful by suicide-bereaved individuals, such as keeping a journal, using bereavement support groups, or structuring each day carefully [23].

In view of the paucity of research work in this area, and the range of patterns of use we identified in our work, there is a need for further qualitative studies to understand misuse of drugs or alcohol after suicide bereavement. Such qualitative accounts would be better contexualised by including objective measures of substance use before and after traumatic loss. They should also investigate whether people would accept support to address hazardous substance use, and from whom. Such work should balance recruitment of ages, genders, and of cultural groups, to explore the range of influences on substance use, separating out cigarette smoking, alcohol use, and a range of different illicit, over-the-counter, and prescribed drugs.

## 5. Conclusions

Our study found that it was common for people to change their use of alcohol and unprescribed drugs after a suicide bereavement, either increasing or decreasing their use. Such patterns were either transient or long-standing pattern, with widespread awareness and concerns about the potential harms. Where individuals were aware of the motivations behind their use of drugs or alcohol, this was primarily to manage emotions and cope with loss, and balance the perceived benefits of drugs or alcohol with their known harms. We identified three main themes, relating to struggles to control use of substances, using substances for a specific purpose, and an attribution of substance misuse to life transitions or peer influences. Our study highlights the care with which clinicians should approach the topic of substance use after traumatic loss, acknowledging the potential for bereaved individuals to perceive some value in transient substance use. It demonstrates the importance of knowing when and how to suggest that those struggling with alcohol or drug use to seek help for this, such that offers of help might be acceptable to those processing a traumatic loss.

## Figures and Tables

**Table 1:** Participants’ Sociodemographic Characteristics.

Characteristic		Total *n* = 346
Gender		*n* (%)
	male	67 (19)
	female	279 (81)
Age		
	18–21	110 (32)
	22–25	94 (27)
	26–30	68 (20)
	31–40	74 (21)
Ethnicity		
	White	316 (91)
	Non-White	30 (9)
Education		
	max GCSE level	6 (2)
	max A level	113 (33)
	max undergraduate degree	132 (38)
	max postgraduate degree	85 (25)
	other	10 (3)

**Table 2:** Higher Order Themes and Subthemes.

	Higher Order Themes	Sub Themes
1	Control over drug or alcohol use	Loss of control over drug or alcohol use Restraint over use of drugs or alcohol
2	Perceived purpose of using drugs or alcohol	Coping with overwhelming thoughts and emotions Honouring the memory of the deceased
3	Attribution of drug or alcohol misuse to external factors	

## Supplementary Materials

The following are available online at https://www.mdpi.com/1660-4601/16/21/4093/s1, supplementary 1: UCL Bereavement Study questionnaire.
